# On the Dynamic Tensile Behaviour of Thermoplastic Composite Carbon/Polyamide 6.6 Using Split Hopkinson Pressure Bar

**DOI:** 10.3390/ma14071653

**Published:** 2021-03-27

**Authors:** Muhammad Ameerul Atrash Mohsin, Lorenzo Iannucci, Emile S. Greenhalgh

**Affiliations:** Department of Aeronautics, Imperial College London, Exhibition Road, London SW7 2AZ, UK; lo.iannucci@imperial.ac.uk (L.I.); e.greenhalgh@imperial.ac.uk (E.S.G.)

**Keywords:** thermoplastic composites, high-performance composites, composite structures, NCF composites, dynamic tensile, split Hopkinson pressure bar, numerical modelling, strain-rate sensitivity

## Abstract

A dynamic tensile experiment was performed on a rectangular specimen of a non-crimp fabric (NCF) thermoplastic composite T700 carbon/polyamide 6.6 specimens using a split Hopkinson pressure (Kolsky) bar (SHPB). The experiment successfully provided useful information on the strain-rate sensitivity of the NCF carbon/thermoplastic material system. The average tensile strength at three varying strain rates: 700, 1400, and 2100/s was calculated and compared to the tensile strength measured from a standardized (quasi-static) procedure. The increase in tensile strength was found to be 3.5, 24.2, and 45.1% at 700, 1400, and 2100/s strain rate, respectively. The experimental findings were used as input parameters for the numerical model developed using a commercial finite element (FE) explicit solver LS-DYNA^®^. The dynamic FE model was validated against experimental gathering and used to predict the composite system’s behavior in various engineering applications under high strain-rate loading conditions. The SHPB tension test detailed in this study provided the enhanced understanding of the T700/polyamide 6.6 composite material’s behavior under different strain rates and allowed for the prediction of the material’s behavior under real-world, dynamic loading conditions, such as low-velocity and high-velocity impact.

## 1. Introduction

There has been a continuous, growing interest in the development and characterization of high-performance thermoplastic composites, primarily in the automotive and aerospace industry. In comparison to thermosetting composites, thermoplastic-based composite materials do not require specific cure schedule and can be processed, heated, and cooled more readily. On top of being recyclable [1], thermoplastics and thermoplastic composites comply with EU directive 2000/53/EC [2]: “the total percentage of preparation for reuse and recycling will be at least 85% of the average weight per vehicle and year”.

The mechanical characterization process of a material is critical in determining its reliability and mechanical performance, particularly under real-world applications under impact conditions, e.g., vehicle collision [3,4], bird strike [5,6], and sports impact [7]. Traditional standardized test procedures are typically performed under quasi-static conditions [8,9,10,11,12,13]. However, if a test specimen is ten millimeters long and is deformed at a loading rate of 1–100 m/s, the strain rate in the specimen is 10^2^–10^4^/s and conventional universal testing machines or load frames are not usually capable of achieving such loading rates. Therefore, high-rate loading conditions are beyond the scope of traditional material testing machines [14].

### 1.1. Characterisation of Dynamic Mechanical Properties of Composites Using Split Hopkinson Pressure Bar

To date, most studies found in the literature that were carried out on the dynamic mechanical properties of composite materials revolved around thermosetting composites [15,16,17,18,19,20] and compressive behavior [20,21,22,23]. Unlike the more commonly used thermoplastic composites that were reinforced with glass fibers [24], short fibers [25], basalt fibers [26,27]; very limited information can be found on in the literature on the dynamic properties, specifically, dynamic tensile properties of continuous carbon fiber-reinforced polymer (CFRP) composites [28,29,30,31], let alone the more recent carbon fiber-reinforced thermoplastic (CFRTP) composites [32]. Furthermore, the majority dynamic analysis of CFRTP focused on unidirectional (UD) fiber orientation [30,33] and very limited information can be found in relation to multidirectional [21,34,35] or bidirectional, especially non-crimp fabric (NCF). Also, in addition to studying the dynamic tensile behavior of UD carbon/thermoset composite, using a servo-hydraulic universal testing machine, Zhang et al. [33] only managed to achieve strain rates from 4 × 10^−5^/s to 160/s. Conversely, split Hopkinson pressure bar (SHPB) allows for much higher, if not the highest possible strain rates. The SHPB technique was implemented on a wide variety of materials, e.g., metals [36], concrete [37], coal [38], and adhesives [39]. Naturally, it was also adapted to the testing composite materials. In the past, this has largely been carried out in compression [40,41]. However, it was adapted for tension [34,35]. Using the SHPB technique, Hou and Ruiz [34] and Gilat et al. [35] reported achieving strain rates of up to 600/s on multidirectional carbon/thermoset composite systems.

Hence, there was a gap in the literature regarding the characterization of the dynamic tensile behavior of an NCF biaxial CFRTP composite, as presented in this study. Given the interest from various industries (as well as the industrial partners of Imperial College), e.g., automotive and aerospace to understand the strain-rate sensitivity of thermoplastic composites, the experimental results presented in this paper are valuable. Moreover, due to recent advancements in and advantages of NCF composites as well as newer NCF-based CFRTPs (such as the one presented in this paper), there is a need for its dynamic mechanical characterization prior to commercialization.

### 1.2. Numerical Model of SHPB Experiment

Numerical model development of SHPB experiments were performed on UD glass fiber-reinforced polymer [42], wood [43], and epoxy [44], but none were found on NCF bidirectional CFTRP. Therefore, there is an opportunity to discuss and describe the development of a numerical model of a CFRTP composite that incorporates the strain-rate dependency of the material.

Based on the results obtained from the SHPB experiment, a numerical model can be developed for the procedure and validated against the experimental findings. To the authors’ knowledge at the time of writing, there has not been a constitutive material model in commercial finite element (FE) that incorporates strain-rate sensitivity of a laminated composite material that can predict its behavior using an energy-based damage model, e.g., based on the translaminar/intralaminar mode I fracture toughness of the material.

The approach proposed in this paper could assist in future numerical model development and should allow for the prediction the structural and mechanical behavior of the component made of similar, strain-rate sensitive materials under comparable loading conditions, and real-world applications e.g., low-velocity impact or high-velocity impact applications [45,46,47].

A numerical model that describes the damage characteristics of laminated composites was reported by Konstantinos et al. [48]. However, the impact energies considered were merely one to two joules. Therefore, knowing the strain-rate sensitivity in this case, was not critical as the mechanical behavior of the composite ought to be very similar to its quasi-static behavior. In comparison, the low-velocity impact energies that were of interest in [45] were 40, 100, and 160 J. Hence, until the dynamic mechanical properties of the composite system at different strain rates have been characterized, it is uncertain whether the (known) quasi-static properties could be implemented in the numerical model.

## 2. Materials and Manufacturing Methods

### 2.1. Material System and Preparation

The CFRTP material system used in this research was a NCF biaxial (0°/90°) T700 (continuous) carbon pre-impregnated with polyamide 6.6 veils (T700/PA6.6) The stitching material of the T700/PA6.6 was also PA6.6. The material was supplied by THERMOCOMP (UK) project [49] partners.

The neat material and mechanical properties of the constituent materials of the composite are shown in Table 1. However, since the material system is proprietary, the mechanical properties of the laminates were obtained by the author using a series of standardized and non-standardized testing described in [47] and listed in Table 2.

The densities of both CFRTP material systems were measured using a pycnometer after being stored in an oven at 40 °C for three days. This was done to completely eliminate moisture. The density of the T700/PA6.6 was measured to be 1485 kg/m^3^.

The fiber-volume-fraction (FVF) of the material system was quantified using thermogravimetric analysis (TGA) to be 52%. The process was documented and described in [55].

### 2.2. Manufacturing Process and Specimen

The laminates of the NCF biaxial 0/90 T700/PA6.6 CFRTP were manufactured using a hand lay-up method and processed using thermoforming method using a laboratory hydraulic press (HÖFER, Taiskirchen, Austria) at 275 °C. Each laminate comprised of two plies of the T700/PA6.6 with the following layup sequence: 0/90/90/0.

The recommended dwell time was 10 min. The heating rate was set to 15 °C/min, following the manufacturer’s recommendation. The recommended pressure for the thermoforming process was 1.5 MPa. The demolding temperature was 25°. The average thickness of the manufactured laminates was measured to be 0.65 mm.

**Figure 1:**
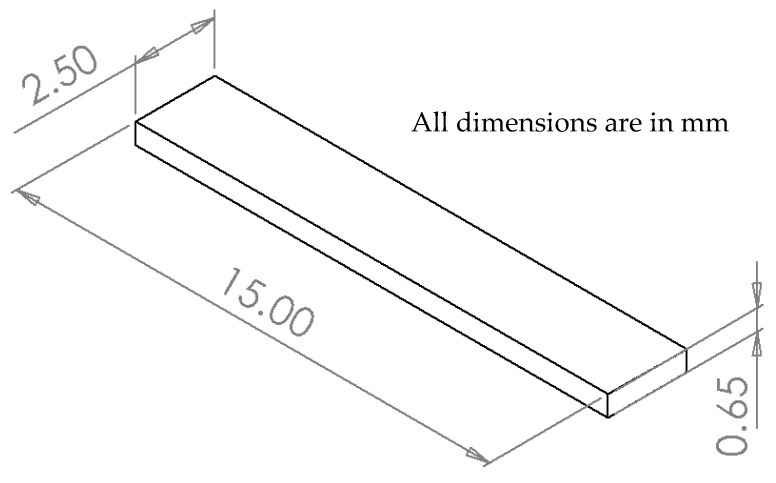
Dimension of the split Hopkinson pressure bar (SHPB) sample for the dynamic tension test (length = 15 mm; width = 2.5 mm; and thickness = 0.65 mm).

**Figure 2:**
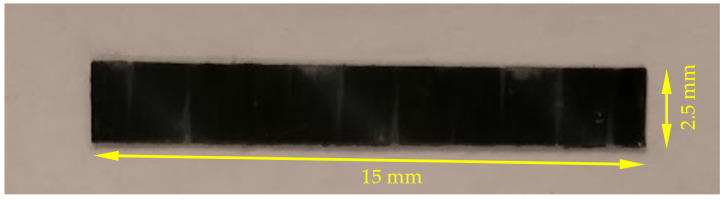
The tensile SHPB test specimen of T700/PA6.6.

The manufactured laminates were cut to form rectangular specimens with the following dimensions, shown in Figure 1. A total of 15 samples (Figure 2) were tested (five for each strain rate).

### 2.3. Experimental Setup

At the time of primary author’s visit to Purdue University, two SHPBs were found to be suitable for the planned experiment: (i) aluminum SHPB and (ii) steel SHPB. Based on the specimen size and the limits of the load cell, it was calculated that the aluminum and steel tension SHPB can achieve strain rates of up to 1500 and 3000/s, respectively. Therefore, it was decided that the dynamic tension experiment would be conducted at three different strain rates: 700, 1400, and 2100/s. The wave dispersion in the experiments was minimized using pulse shaping techniques, where a small piece of material was placed on the end of the incident bar, known as the ‘pulse shaper’. Based on the information provided to the author, the pulse shaper material used during the experiment was made of copper.

**Figure 3:**
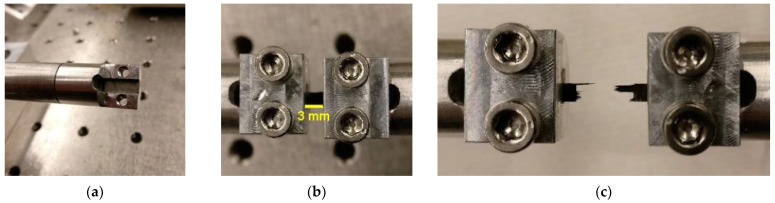
Image of: (**a**) the fixture of the aluminum SHPB that accommodates the specimen, (**b**) the specimen mounted onto the SHPB with a gauge length of 3 mm (prior to the test), and (**c**) tensile fracture of the specimen.

The rectangular sample was placed into a fixture (Figure 3a). This cylindrical fixture consists of a flat rectangular groove where a (rough) section of a file was glued onto. The surface roughness of the file provides the necessary friction required to hold the specimen in place. This fixture was then screw into the bar (SHPB) via a threaded connection. Figure 3b indicates how the specimen was held in the fixture and the 3 mm gauge length. Figure 3c illustrates the typical tensile fracture of the specimen after the test.

## 3. Data Reduction

### 3.1. Energy Calculation

All of the Equations (1)–(49), which are presented here were obtained from Chen and Song [14]. Based on the one-dimensional wave propagation theory, the dynamic stress-strain response of the specimen can be derived as follows

(1) $$\sigma =E\frac{\partial u}{\partial y},$$

where E is the material Young’s modulus. Hence, the one-dimensional equation of motion is

(2) $$\frac{\partial \sigma }{\partial y}=\rho \frac{\partial^{2}u}{\partial t^{2}},$$

where u is the displacement and ρ is the density of the material. Combining Equations (1) and (2)

(3) $$E\frac{\partial^{2}u}{\partial y^{2}}=\rho \frac{\partial^{2}u}{\partial t^{2}},$$

Therefore, the equation of motion in the bar for a wave of infinite wavelength along direction y can be written as follows

(4) $$C_{0}{}^{2}\frac{\partial^{2}u}{\partial y^{2}}=\frac{\partial^{2}u}{\partial t^{2}},$$

where $C_{0}$ is the wave velocity. Comparing Equations (3) and (4), $C_{0}$ can be rewritten as

(5) $$C_{0}=\sqrt{\frac{E}{\rho }},$$

**Figure 4:**
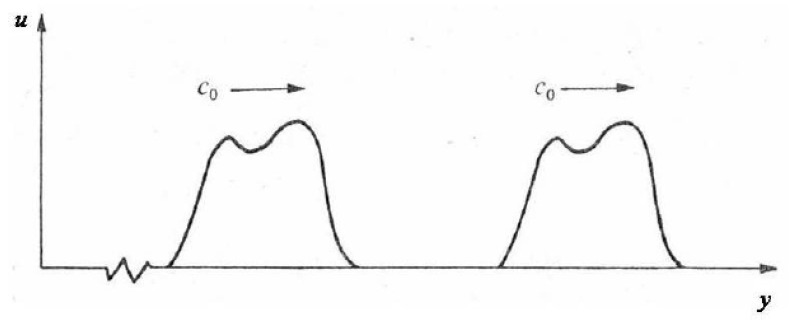
Wave propagation in the positive y-direction, where u is the diplacement and $C_{0}$ is the wave velocity.

The D’Alembert’s equation for wave equation (Equation (4)) is

(6) $$u\left(y,t\right)=f\left(y-C_{0}t\right)+g\left(y+C_{0}t\right),$$

where the function $f\left(y-C_{0}t\right)$ and $g\left(y+C_{0}t\right)$ correspond to a wave propagating in the positive and negative y-direction, respectively. If the wave propagates only in the positive y-direction (Figure 4), Equation (6) reduces to

(7) $$u\left(y,t\right)=f\left(y-C_{0}t\right),$$

and if the wave propagates only in the negative y-direction, Equation (6) reduces to

(8) $$u\left(y,t\right)=g\left(y+C_{0}t\right),$$

Considering the wave travels in the positive y-direction, using Equation (7), the strain in the bar can written as

(9) $$\varepsilon_{b}\left(y,t\right)=\frac{\partial u}{\partial y}=f^{\prime }\left(y-C_{0}t\right),$$

and Hooke’s law can be applied to determine the stress as follows

(10) $$\sigma_{b}\left(y,t\right)=E\varepsilon_{b}\left(y,t\right)=Ef^{\prime }\left(y-C_{0}t\right),$$

and force, F, on the cross-sectional area of the bar, $A_{0},$ can be expressed as

(11) $$F=A_{0}\sigma_{b}\left(y,t\right)=A_{0}E\varepsilon_{b}\left(y,t\right)=A_{0}Ef^{\prime }\left(y-C_{0}t\right)\;,$$

Differentiating u(y,t) with respect to time, t, yields

(12) $$v\left(y,t\right)=\frac{\partial u}{\partial y}=-C_{0}f^{\prime }\left(y-C_{0}t\right)\;,$$

where v(y,t) is the particle velocity in the y-direction with respect to time, t. Combining Equations (9) and (13), the particle velocity

(13) $$v\left(y,t\right)=-C_{0}\varepsilon_{b}\;,$$

and the ratio of the applied force on the bar and particle velocity, v, is the impedance, Z, where

(14) $$Z=\frac{F}{v}=-\frac{A_{0}E}{C_{0}}\;,$$

Based on Equation (14), by substituting the Young’s modulus, E, the impedance, Z, can be expressed as

(15) $$Z=\frac{F}{v}=A_{0}\rho C_{0}\;,$$

**Figure 5:**
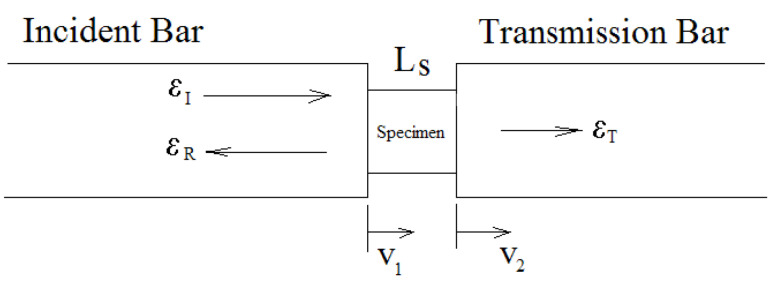
Testing section of the SHPB.

On the assumption that the stress waves propagate in both the transmission bars without dispersion (where the pulses recorded by strain gauge represent those at the bar ends, in contact with the specimen), one-dimensional stress wave theory relates the particle velocities at both ends of the specimen to the three strain pulses recorded as illustrated in Figure 5.

Thus, $v_{1}$ and $v_{2}$ can be expressed as

(16) $$v_{1}=C_{B}\left(\varepsilon_{I}-\varepsilon_{R}\right),$$

(17) $$v_{1}=C_{B}\varepsilon_{T}\;,$$

where the subscripts, I, R, and T correspond to the incident, reflected, and transmitted pulses, respectively. The average engineering strain rate, $\dot{\varepsilon },$ and strain, ε, in the specimen can be written as:

(18) $$\dot{\varepsilon }=\frac{v_{1}-v_{2}}{L_{S}}=\frac{C_{B}}{L_{S}}\left(\varepsilon_{I}-\varepsilon_{R}-\varepsilon_{T}\right)\;,$$

(19) $$\varepsilon =\int_{0}^{t}\dot{\varepsilon }dt=\frac{C_{B}}{L_{S}}\int_{0}^{t}\left(\varepsilon_{I}-\varepsilon_{R}-\varepsilon_{T}\right)dt\;,$$

where $L_{S}$ is the initial length of the specimen. The stresses at both ends of the specimen can be calculated using the elastic relations

(20) $$\sigma_{1}=\frac{A_{B}}{A_{S}}E_{B}\left(\varepsilon_{I}+\varepsilon_{R}\right),$$

(21) $$\sigma_{2}=\frac{A_{B}}{A_{S}}E_{B}\varepsilon_{T}\;,$$

where $A_{B}$ and $A_{S}$ are the cross-sectional areas of the bar and test specimen, respectively. $E_{B}$ is the Young’s modulus of the material of the bar. The specimen is assumed to be under stress equilibrium in SHPB experiment. Therefore, this assumption must be satisfied in the dynamic characterization of the material properties. Hence, the sample deforms uniformly and the response obtained is the average response over its total volume. This is regarded as a good representative of the point-wise valid material behavior. So,

(22) $$\sigma_{1}=\sigma_{2}\;,$$

and from Equations (20) and (21),

(23) $$\varepsilon_{I}+\varepsilon_{R}=\varepsilon_{T}\;,$$

and Equations (18), (19), and (21) can be simplified as

(24) $$\dot{\varepsilon }=-2\frac{C_{B}}{L_{S}}\varepsilon_{R}\;,$$

(25) $$\varepsilon =-2\frac{C_{B}}{L_{S}}\int_{0}^{t}\varepsilon_{R}dt\;,$$

(26) $$\sigma =\frac{A_{B}}{A_{S}}E_{B}\varepsilon_{T}\;,$$

The (average) elastic strain energy, $E_{I}$, associated with the incident wave can be calculated as follows using the incident strain, $\varepsilon_{I}$

(27) $$E_{I}=V_{I}\int_{0}^{\varepsilon_{I}}\sigma d\varepsilon \;,$$

where $V_{I}$ is the deformed volume in the incident bar. Nonetheless, during the stress wave propagation, only a section of the incident bar is involved in the elastic deformation following the incident pulse. Thus, the deformed volume, $V_{I},$ in the incident bar is dependent on the loading duration and the cross-sectional area of the bar. This can then be expressed as follows

(28) $$V_{I}=A_{0}C_{0}T,$$

where T is the loading duration, which can be described by the following equation

(29) $$T=\frac{2L}{C_{st}}\;,$$

where $C_{st}$ is the elastic wave speed of the striker material. The stress of a linearly elastic bar, σ

(30) $$\sigma =E_{B}\varepsilon \;,$$

Therefore, the elastic strain energies associated with the reflected, $E_{R}$, and transmitted waves, $E_{T}$, can be expressed as follows

(31) $$E_{R}=\frac{1}{2}A_{B}C_{B}E_{B}T\varepsilon_{R}{}^{2}\;,$$

(32) $$E_{T}=\frac{1}{2}A_{B}C_{B}E_{B}T\varepsilon_{T}{}^{2}\;,$$

Hence, the influence of elastic strain energy in the bars in relation to the specimen deformation can be calculated as

(33) $$\delta_{E}=E_{I}-E_{R}-E_{T}=\frac{1}{2}A_{B}C_{B}E_{B}T\left(\varepsilon_{I}{}^{2}-\varepsilon_{R}{}^{2}-\varepsilon_{T}{}^{2}\right)\;,$$

or simplified as

(34) $$\delta_{E}=-A_{B}C_{B}E_{B}T\varepsilon_{R}\varepsilon_{T}\;,$$

when the specimen is under dynamic stress equilibrium. The kinetic energy of the incident bar, $K_{I}$, after the incident wave propagates can be written as

(35) $$K_{I}=\frac{1}{2}mv_{I}{}^{2}\;,$$

where m is the mass and v is the particle velocity of the deformed section of the incident bar. The mass, m, and the velocity, $v_{I}$, can be gathered from

(36) $$m=\rho_{B}A_{B}C_{B}T\;,$$

(37) $$v_{I}=C_{B}\varepsilon_{I}\;,$$

Using Equations (36) and (37), Equation (35), can be rewritten as

(38) $$K_{I}=\frac{1}{2}\rho_{B}A_{B}C_{B}{}^{3}T\varepsilon_{I}{}^{2}\;,$$

and similarly, the kinetic energy corresponding to the reflected, $K_{R}$, and transmitted, $K_{T}$, pulses can be expressed as

(39) $$K_{R}=\frac{1}{2}\rho_{B}A_{B}C_{B}{}^{3}T\varepsilon_{R}{}^{2}\;,$$

(40) $$K_{T}=\frac{1}{2}\rho_{B}A_{B}C_{B}{}^{3}T\varepsilon_{T}{}^{2}\;,$$

Assuming that the specimen is in stress equilibrium, the relationship between the specimen deformation and kinetic energy can be written as

(41) $$\delta_{KE}=-\rho_{B}A_{B}C_{B}{}^{3}T\varepsilon_{R}\varepsilon_{T}\;,$$

Considering linear elastic bars, the Young’s modulus of the bar, $E_{B}$, can also be expressed as

(42) $$E_{B}=\rho_{B}C_{B}{}^{2}\;,$$

and Equation (41) will now become

(43) $$\delta_{K}=-A_{B}E_{B}C_{B}T\varepsilon_{R}\varepsilon_{T}\;,$$

where Equation (43) is now in the same form as Equation (34). Based on the assumption of perfectly plastic response, the deformation energy can be simplified as

(44) $$E_{S}=A_{S}L_{S}\sigma_{y}\varepsilon_{p}\;,$$

where $A_{S}$ and $L_{S}$ are the initial cross-sectional area and length of the sample, respectively. $\sigma_{y}$ is the yield strength (expressed in Equation (45)) and $\varepsilon_{p}$ is the plastic strain of the specimen (described by Equation (46)).

(45) $$\sigma_{y}=\frac{A_{B}}{A_{S}}E_{B}\varepsilon_{T}\;,$$

(46) $$\varepsilon_{p}=\varepsilon T=-2\frac{C_{B}}{L_{S}}\varepsilon_{R}T\;,$$

Now, Equation (44) can be written as

(47) $$E_{S}=-A_{B}E_{B}C_{B}T\varepsilon_{R}\varepsilon_{T}=2\sigma_{E}=2\sigma_{K}\;,$$

where it can be concluded that the energy coming from the elastic strain energy in the bars yields half of the energy necessary for the plastic deformation and the remaining half is contributed by the kinetic energy.

### 3.2. Stress Calculation from Raw Signals

In the SHPB experiment, the raw signals consisting of the incident, reflected, and transmitted pulses were processed and plotted. The properties of both aluminum and steel SHPB are listed in Table 3.

The gauge factor, $G_{F}$ of the strain gauge mounted on the bar is defined as [14]

(48) $$G_{F}=\frac{\Delta R}{R}\frac{1}{\varepsilon }\;,$$

where ΔR is the change is resistance, R. The relationship between the voltage output, $U_{O}$, voltage input, $U_{I}$, and strain, ε, is described by the following equation [14]

(49) $$\varepsilon =\frac{2U_{O}}{G_{F}U_{I}}\;,$$

## 4. Experimental Results and Discussion

### 4.1. Relationship between Tensile Strength and Strain Rate

**Figure 6:**
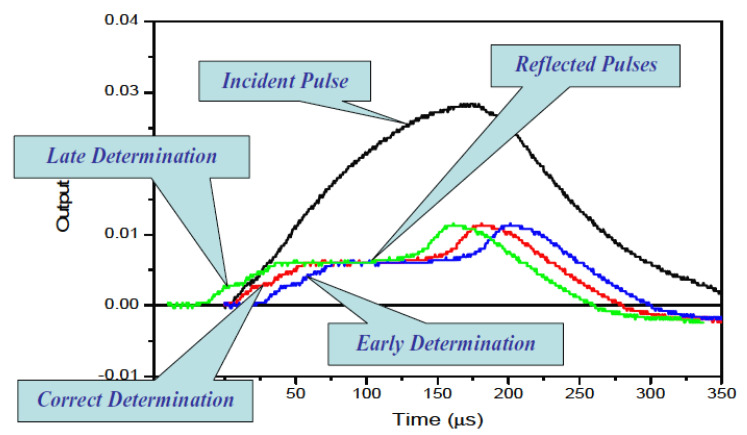
Determination of reflected pulse, the correct and incorrect method. Reprinted with permission from Springer, Split Hopkinson (Kolsky) Bar by Chen and Song (2011) [14].

**Figure 7:**
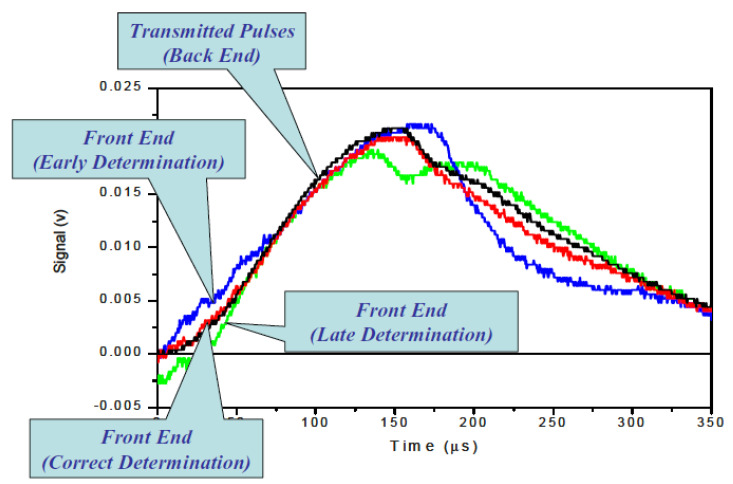
Determining the stress equilibrium, the correct and incorrect method. Reprinted with permission from Springer, *Split Hopkinson (Kolsky) Bar* by Chen and Song (2011) [14].

**Figure 8:**
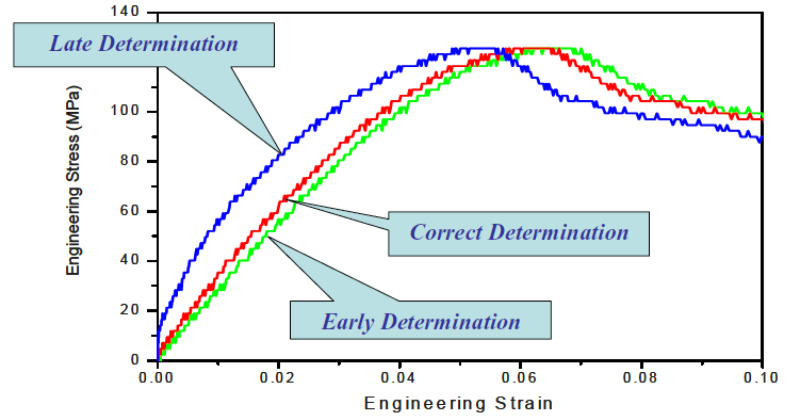
Determination of stress-strain curves, the correct and incorrect method. Reprinted with permission from Springer, *Split Hopkinson (Kolsky) Bar* by Chen and Song (2011) [14].

Figure 6 illustrates the correct and incorrect starting-point determinations of the reflected pulse for a certain incident pulse. Given that the experiment was correctly designed, the accurate determination of the starting point corresponds to the stress equilibrium across the specimen length. Incorrect starting points of the reflected pulse will result in erroneous calculation of the force at the initial (front) end of the specimen, which will provide a wrong assessment of the dynamic stress equilibrium (depicted in Figure 7). Such incorrect determination of the starting point leads to erroneous stress–strain curves (shown in Figure 8).

**Figure 9:**
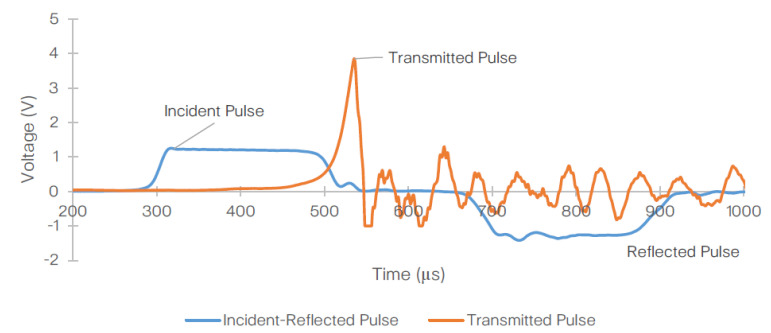
Representative raw signals obtained from one of the 2100/s strain rate following the SHPB experiment conducted on T700/PA6.6 specimen.

**Figure 10:**
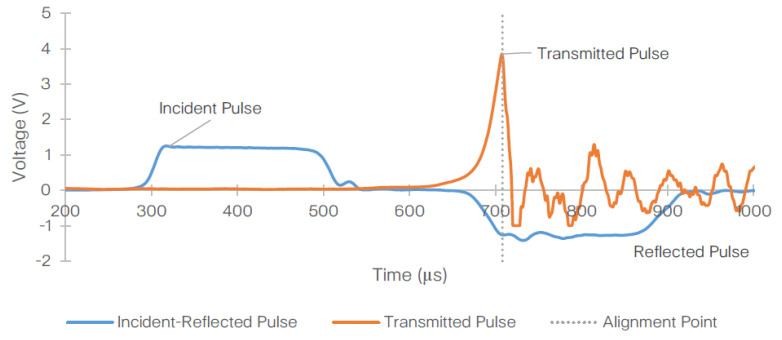
Representative plot of the corrected (aligned) raw signals obtained from one of the 2100/s strain rate following the SHPB experiment conducted on T700/PA6.6 specimen.

Figure 9 shows the representative stress wave signals obtained from the SHPB test at 2100/s strain rate. Evidently, the transmitted and reflected pulses are misaligned. Therefore, prior to performing the calculations detailed in Section 3.1 and Section 3.2, the reflected and transmitted waves must be aligned (as shown in Figure 10), where the peak of the transmitted pulse is now matching the (reverse) peak of the reflected pulse. The data obtained throughout the test reached saturation beyond peak force. The sampling rate on the data acquisition system was 0.2 µs.

Using Equations (48) and (49) and those discussed in Section 3.1 and Section 3.2, the stress and strains were calculated and plotted for each strain rate.

**Figure 11:**
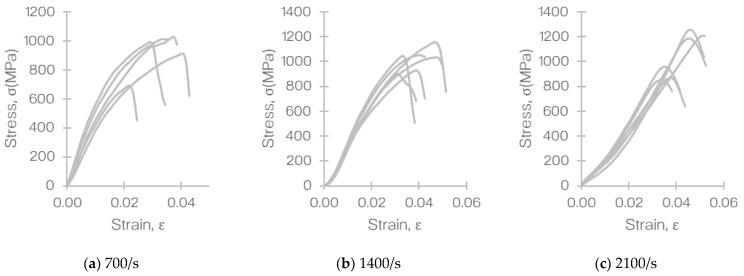
Stress–strain plots obtained from the SHPB tensile test of T700/PA6.6 at different strain rates: (**a**) 700, (**b**) 1400, and (**c**) 2100/s.

The stress-strain relationship observed in the experiments at the three different strain rates is illustrated in Figure 11. Based on this, the average strengths were calculated and presented in Table 4. The average tensile strengths at 700, 1400, and 2100/s were 950 (CV = 3.3%), 1140 (CV = 7.8%), and 1332 MPa (CV = 8.5%), respectively. The variation seen in the experiment was predominantly caused by the marginal discrepancy in the quality of the coupon samples that were cut from different areas of the laminated composite panel. However, the CVs reported here were found to be comparable to variation seen in the standardized and non-standardized tests when characterizing the material [47].

**Figure 12:**
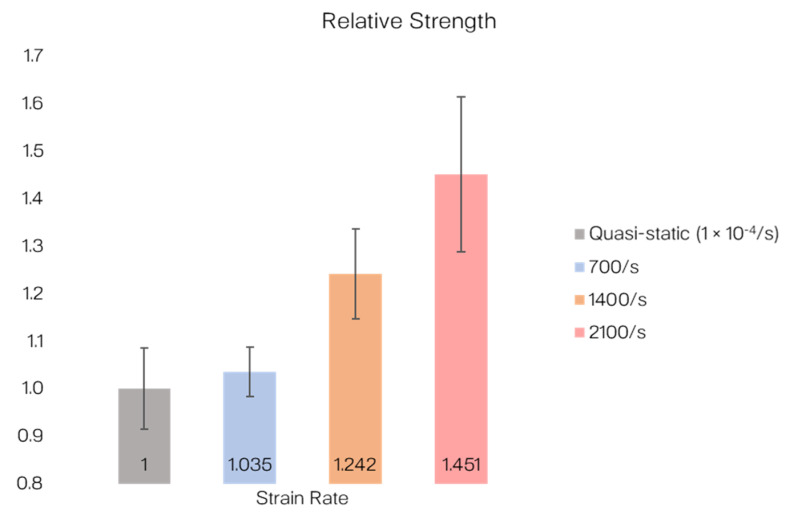
Quasi-static tensile strength of T700/PA6.6 versus dynamic tensile strengths (normalized to the quasi-static tensile strength).

In comparison to the quasi-static results (obtained from a standardized test [56] in [47]), the average strengths obtained from the dynamic SHPB tests were higher: +3.5% at 700/s, +24.2% at 1400/s, and +45.1% at 2100/s. Figure 12 illustrates the relative tensile strengths of the T700/PA6.6 specimens compared to the standardized quasi-static test.

It must be noted that a nonlinearity (denoted by the curvature) in the stress–strain behavior of the T700/PA6.6 was observed in Figure 11 and found to be more dominant at higher strain rates, which was predicted. This nonlinearity was believed to be contributed by the following:

- *Geometrical effects* of the fixtures used and threaded connection between the fixtures and the bar
- *Pulse dispersion* that was caused by the wave that propagates through the bar, which had to travel through the threads of the fixture, the fixture, and then the specimen
- *Viscoelasticity of the interface* between the fixture and the file as the file was glued to the fixture using an epoxy-based glue (which is viscoelastic)

### 4.2. Experimental Conclusion

Therefore, given the nature of the novel SHPB test setup and its limitations, the apparent (slight) nonlinearity of the stress-strain curves was expected. Moreover, it was physically and mechanically natural for the (three) factors contributing towards the nonlinearity to be more pronounced at higher strain rates.

In conclusion, it was found that the T700/PA6.6 CFRTP system was influenced by the strain-rate effect, where the tensile strength increases as the strain rate increases. Although the exact information on the strain-rate dependency of this specific fiber-matrix combination, the results were consistent with a wide variety of similar thermoplastic composite laminates [57], where significant nonlinear and strain rate dependency behavior have been observed.

## 5. Finite Element Model of the Tension SHPB Experiment

### 5.1. Model Description

**Figure 13:**
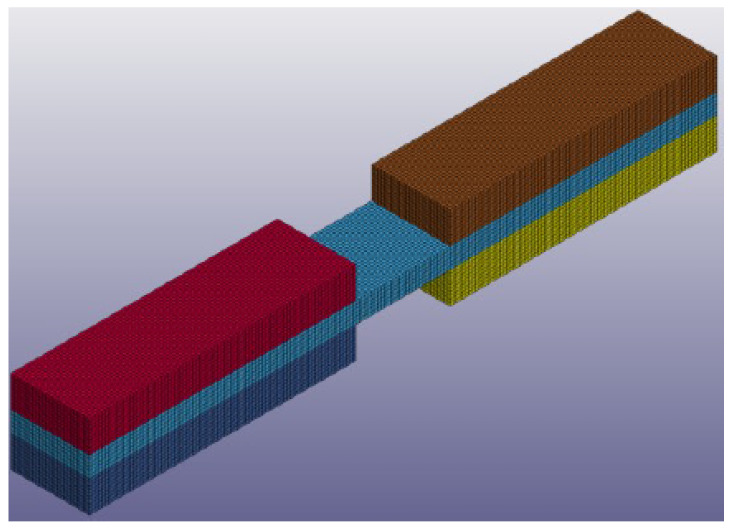
Finite element (FE) model of the tension SHPB specimen in LS-DYNA^®^.

To simulate the SHPB experiment, a dynamic finite element (FE) model was developed in LS-DYNA^®^ R8.1.0 (R8.105896) (LSTC, Livermore, CA, USA). For simplification, the model was composed of a section of bespoke SHPB (rectangular) fixture that was in contact with the specimen and the specimen itself (Figure 13), not the entire (cylindrical) SHPB. The boundary conditions and specimen constraints were set to mimic the actual test.

**Figure 14:**
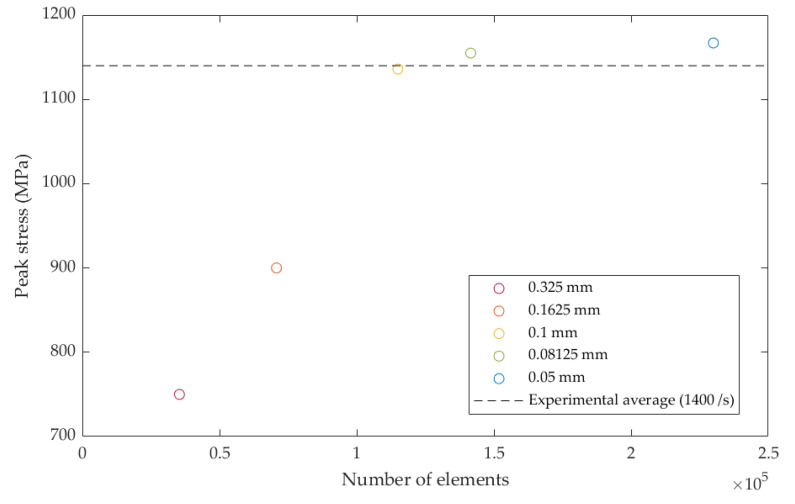
Mesh dependency study indicating that the 0.1 mm is the optimal element size.

The interaction between the section of the fixture that held the specimen and the specimen was modelled using LS-DYNA^®^ TIED_SURFACE_TO_SURFACE (LSTC, Livermore, CA, USA) contact algorithm. The material card used to predict the T700/PA6.6 behavior was the energy-based MAT_262-LAMINATED_FRACTURE_DAIMLER_CAMANHO (LSTC, Livermore, CA, USA). The material card was populated using a combination of the quasi-static (Table 2) and dynamic mechanical properties (Table 4). For simplification, other (basic) mechanical properties in the material model description, e.g., Young’s modulus, in-plane shear, etc., were also scaled using the same factor obtained in the SHPB experiment. Following the mesh convergence study (Figure 14), the optimized element size was determined to be 0.1 mm (100 µm) and this was set throughout the model. The model consisted of 115,000 of solid elements. Using a quad core, hyper-threaded Intel^®^ Core i7-4930 MX (Intel^®^, Santa Clara, CA, USA), a simulation took approximately 4–8 h to complete.

The strain rates that were achieved in the SHPB experiment were replicated in the model. To achieve this, the fixture was prescribed with the (average) initial velocity observed in the experiment and the same inertia (mass) of the SHPB setup (either the aluminum or steel bar) with respect to the desired strain rate. The input parameters used to describe the model in LS-DYNA^®^ can be found in Table 5.

### 5.2. Finite Element Analysis versus Experimental Results

**Figure 15:**
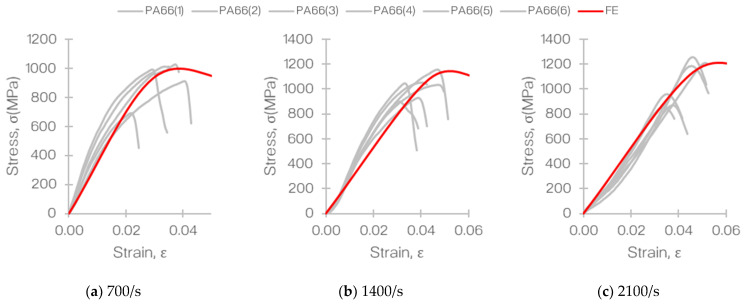
Comparison of stress-strain plots of T700/PA6.6 at different strain rates: (**a**) 700, (**b**) 1400, and (**c**) 2100/s obtained from the experiments and finite element analysis (FEA).

The experimental results obtained and discussed in Section 4.1. were used to validate the numerical model that was developed in LS-DYNA^®^. Therefore, the stress and strain outputs from the model were extracted and compared against the experimental gathering. This is depicted in Figure 15.

Based on the stress–strain relationship seen in Figure 15, it was evident that the results obtained from the finite element analysis (FEA) were in good agreement with the experimental findings. Clearly, the nonlinearity observed in the experiments was not replicated in the FEA as the entire SHPB assembly was not modelled.

Based on the author’s knowledge, to date, there has not been a constitutive model (in a commercial FE solver) with strain-rate sensitivity description that can accurately predict the dynamic behavior of multidirectional laminated composite material. The FE model detailed in this paper does not claim to fully address that, but since the model was validated for three different strain rates, therefore, to a certain extent, the approach (SHPB experiments with FEA) can be used to predict a laminated composite material behavior at a specific strain rate using the SHPB experimental data at that strain rate. In this way, the methodology can be implemented to assess a laminated composite material’s behavior in real-world applications associated with the range of strain rates seen here, such as low-velocity impact (LVI) and high-velocity impact (HVI).

In fact, the data and results presented in this paper were successfully used to model the prediction of LVI [45] and HVI [46] performance of the T700/PA6.6 with FVF = 52%.

## 6. Conclusions

In this study, the goal was to measure the dynamic tensile properties of the NCF biaxial T700/PA6.6 CFRTP composite material system. To achieve this, the specimens of the material were tested using SHPB in tension at three different strain rates: 700, 1400, and 2100/s.

The data gathered from the SHPB experiments described in this paper provided meaningful information on the strain-rate dependency of the laminated thermoplastic composite material system. The T700/PA6.6 carbon/thermoplastic composite material system was found to be strain-rate dependent (sensitive). Therefore, the tensile properties, e.g., tensile strength, increased at higher strain rates. The standard deviations, presented in terms of coefficient of variation (CV), observed from the experimental data sets were primarily due to the nature of manufacturing quality of the composite panel. This was found to be similar to the variations seen in the standardized and non-standardized tests performed on the material.

The nonlinearity of the stress–strain behavior of the material in the experiment was found to be influenced by the following: (i) geometrical effects of the experimental assembly, (ii) dispersion of the pulse of the propagated waves, and (iii) viscoelasticity of the interface within the fixture.

An FE model was also successfully developed to simulate the SHPB tensile test and was validated against the experimental data. The numerical model predictions were in good agreement with the experimental data sets. Hence, the study shows that by knowing the strain-rate sensitivity of the material, the quasi-static constitutive model can be partially modified to replicate the material’s dynamic behavior at a given strain rate. Specifically, this was done by incorporating the quasi-static mechanical properties and scaling them to the dynamic mechanical properties.

The experimental results and the FE model in this study were proven to be valuable as they allow for the prediction of the material’s behavior under LVI [45] and HVI [46] using the correct implementation of the material’s mechanical properties with respect to the strain rates, under such dynamic loading conditions.

The methodology that was presented in this research was proven to be useful for the authors, and therefore, could potentially assist future researchers, scientists, and engineers in understanding and modelling the dynamic behavior of their composite materials of choice.

## Figures and Tables

**Table 1:** Mechanical properties of neat PA6.6 and T700 fiber.

References	PA6.6	T700 Fiber
	[47,50,51]	[52]
Density (kg/m^3^)	1170	1800
Tensile strength, ultimate (MPa)	~70	4900
Tensile modulus (GPa)	2.5	230
Elongation at break (%)	53.9	2.1
Mode I fracture toughness, $G_{IC}$ (kJ/m^2^)	0.2	-

**Table 2:** Quasi-static mechanical properties of T700/PA6.6 with a fiber-volume-fraction of 52% [47,53,54].

Mechanical Properties	Material: T700/PA6.6
Tensile Young’s modulus (GPa)	65
Compressive Young’s modulus (GPa)	69
Tensile strength (MPa)	918
Compressive strength (MPa)	461
In-plane shear modulus (GPa)	3.2
In-plane shear stress at 5% (MPa)	52
Mode I interlaminar fracture toughness, $G_{IC}$ (kJ/m^2^) [53]	1.50
Mode II interlaminar fracture toughness, $G_{IIC}$ (kJ/m^2^) [47]	1.94
Translaminar tensile fracture toughness, $G_{Ic}^{T}$ (kJ/m^2^)	235

**Table 3:** Properties and specification of the aluminum and steel SHPB.

Bar Type	Aluminum	Steel
Gauge factor, $G_{F}$	2.08	151
Wave speed of the bar, $C_{B}$ (m/s)	5100	5050
Tensile strength (MPa)	500–1500	1000–3000

**Table 4:** Summary of the dynamic tensile properties obtained from the SHPB experiment of the T700/PA6.6.

	Test Type			
	Dynamic SHPB			Quasi-Static
	700/s	1400/s	2100/s	
Average strength (MPa)	950	1140	1332	918
Coefficient of variation, CV (%)	3.3	7.8	8.5	8.3
Change vs. quasi-static (%)	+3.5	+24.2	+45.1	-

**Table 5:** Parameters used to describe the mechanical properties of the T700/PA6.6 CFRTP composite [47,53] and the contact surface.

Decoupled in-Plane Mechanical Properties	
Longitudinal Young’s modulus, in the fibre direction, $E_{a}$ (GPa)	129
Transverse Young’s modulus, $E_{b}$ (GPa)	5.0
Shear modulus, $G_{ab}$ (GPa)	3.2
Longitudinal tensile strength, $X_{t}$ (MPa)	2400
Longitudinal compressive strength, $X_{c}$ (MPa)	1500
Transverse tensile strength, $Y_{t}$ (MPa)	156
Transverse compressive strength, $Y_{c}$ (MPa)	189
Shear strength, $S_{ab}$ (MPa)	110
**In-plane fracture toughness**	
Translaminar fracture toughness in compression, $G_{Xc}$ (kJ/m^2^)	350
Translaminar fracture toughness in tension, $G_{Xt}$ (kJ/m^2^)	470
Transverse fracture toughness in compression, $G_{Yc}$ (kJ/m^2^)	4.0
Transverse fracture toughness in tension, $G_{Yt}$ (kJ/m^2^)	4.0
**Contact (cohesive) surface properties**	
Normal failure stress, NFLS (MPa)	60
Shear failure stress, SFLS (MPa)	120
Exponent in the damage model XMU, PARAM	1.8
Mode I interlaminar fracture toughness, $G_{IC}$ (kJ/m^2^) [53]	1.50
Mode II interlaminar fracture toughness, $G_{IIC}$ (kJ/m^2^) [47]	1.94

## Data Availability

Not applicable.

## Nomenclature

$E_{a}$	Longitudinal Young’s modulus, in the fiber direction (Pa)
$E_{b}$	Transverse Young’s modulus (Pa)
$G_{F}$	Gauge factor
$G_{IC}$	Mode I fracture toughness (kJ/m^2^)
$G_{IIC}$	Mode II fracture toughness (kJ/m^2^)
$G_{Ic}^{T}$	Translaminar fracture toughness (kJ/m^2^)
$G_{Xc}$	Translaminar fracture toughness in compression
$G_{Xt}$	Translaminar fracture toughness in tension
$G_{Yc}$	Transverse fracture toughness in compression
$G_{Yc}$	Transverse fracture toughness in compression
$G_{ab}$	Shear modulus (Pa)
$S_{ab}$	Shear strength (Pa)
$U_{I}$	Voltage input
$U_{O}$	Voltage output
$X_{c}$	Longitudinal compression strength (Pa)
$X_{t}$	Longitudinal tensile strength (Pa)
$Y_{c}$	Transverse compression strength (Pa)
$Y_{t}$	Transverse tensile strength (Pa)
$\delta_{E}$	Elastic strain energy (J)
$\dot{\varepsilon }$	Strain rate
CFRTP	Carbon fiber-reinforced thermoplastic
FE	Finite element
FEA	Finite element analysis
FVF	Fiber-volume-fraction
HVI	High-velocity impact
LVI	Low-velocity impact
NCF	Non-crimp fabric
NFLS	Normal failure stress (Pa)
PA	Polyamide
PARAM	Exponent in the damage model XMU
SFLS	Shear failure stress (Pa)
SHPB	Split Hopkinson pressure bar
TGA	Thermogravimetric analysis
A	Cross-sectional area (m^2^)
C	Wave velocity (m/s)
E	Young’s modulus (Pa)
F	Force (N)
K	Kinetic energy (J)
L	Length of specimen (m)
T	Loading duration (s)
V	Deformed volume (m^3^)
Z	Impedance (Ns/m)
m	Mass (kg)
t	Time (s)
u	Particle displacement (m)
v	Particle velocity (m/s)
ε	Strain
ρ	Density (kg/m^3^)
σ	Stress (Pa)
